# Effect of Amino Acid Substitutions at Site F397 on the Kinetic and Thermostability Properties of *Paenibacillus polymyxa* β-Glucosidase B

**DOI:** 10.17912/micropub.biology.002061

**Published:** 2026-03-31

**Authors:** Alyssa Yanes, Marianne Gutierrez Santos, Isabella Chiang, Jaime Mayoral

**Affiliations:** 1 Department of Biological Sciences, Florida International University, Miami, FL, US

## Abstract

β-Glucosidase B (BglB) hydrolyzes β‑glycosidic bonds in complex carbohydrates, playing an important role in global carbon cycling, and diverse biofuel and biotechnological applications. This study systematically evaluated the functional and structural role of residue F397 by generating and characterizing all 19 possible amino acid substitutions. Hydrophobic and neutral substitutions expressed efficiently and retained catalytic efficiency and substrate affinity comparable to wild-type, whereas charged or polar substitutions reduced BglB expression and structural stability. Kinetic analyses showed minimal effects on K _M_ , while thermal stability was generally decreased. These results indicate that F397 primarily contributes to local structural stability rather than catalysis.

**Figure 1. Effect of Amino Acid Substitutions on site F397 of Paenibacillus polymyxa β-Glucosidase B:** **(A)** PyMOL visualization of β‑Glucosidase B (PDB: 2JIE) highlighting residue F397, showing its peripheral position relative to the catalytic center. **(B)** Predicted Foldit energy scores for expressed and non‑expressed F397 mutants; lower energy values correspond to greater predicted stability. **(C)** Multiple sequence alignment at position F397 of 50 β-Glucosidase B bacterial homologs generated using Jalview. **(D)** β-Glucosidase B recombinant expression of F397 mutants grouped by amino‑acid physicochemical class: charged (positive and negative), polar&nbsp; (uncharged), hydrophobic, and aromatic (amino acids with aromatic R-group). ( **E** ) Expression levels of F397 variants grouped by Foldit predicted energy score quartiles. Quartile boundaries were: Q1 (-1033.911 – -1079.353), Q2 (-1079.353 – -1082.524), Q3 (-1082.524 – -1083.972), and Q4 (-1083.972 – -1089.166). **(F)** Catalytic efficiency (K _cat_ /K _M_ ) of wild-type and F397 variants, with most substitutions reducing activity; F397M shows the highest remaining activity among the variants. **(G)** T _50_ thermal‑unfolding temperatures of wild-type and F397 variants. Expressed mutations decrease thermal stability. F397A, F397N, and F397L retain T _50_ values closest to wild-type. For all panels, bars represent the mean, with error bars indicating ± standard deviation (SD).

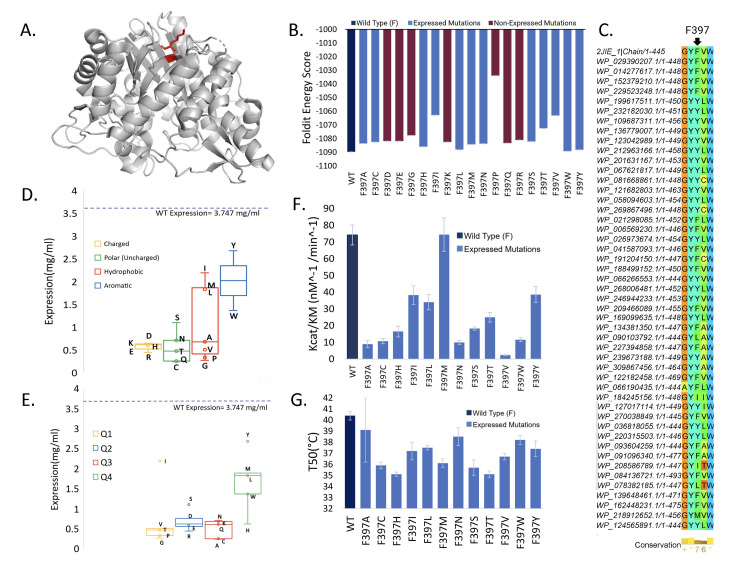



## Description

Enzymes catalyze biochemical reactions in all living organisms, providing stability and enhancing the efficiency of metabolic processes. β-Glucosidase B (BglB) plays a key role in the degradation of cellulose making it crucial for the function of various microorganisms like fungi, yeasts, and bacteria (Goswami et al., 2016). Its involvement in biological processes like carbon cycling also positions BglB as a key target for advances in biofuel production and biotechnology (Zang, 2018).&nbsp;

The Design2Data (D2D) program is a multi-institutional network using student generated data to conduct research on protein and enzyme mutations, aiming to improve predictive enzyme models and algorithms studying β-Glucosidase B (Vater et al., 2021). This study was conducted in the context of the D2D program and focused on evaluating the functional and structural relevance of the amino acid site F397 in BglB. Site F397 was systematically mutated to all other 19 possible amino acids to assess its contribution to the enzyme structure and catalysis. F397 does not seem to form hydrogen bonds to surrounding amino acids and is located towards the periphery of the protein, away from the catalytic center ( **Fig. 1A** ), suggesting it may tolerate substitutions without severely affecting structural stability or function. An alignment of fifty bacterial BglB sequences revealed that F397 has a conservation score of approximately 70%; natural occurring mutations found in different bacterial species were tyrosine, leucine, methionine, and isoleucine **( Fig. 1B )** . These residues are hydrophobic, neutral, and relatively large, suggesting a natural preference for these physiochemical properties at this site.

To investigate the effect of all possible 19 amino acid mutations at F397, expression levels (mg/mL), K _M_ (mM), k _cat_ /K _M_ (nM ^-1^ /min ^-1^ ), and T _50_ (°C) values were assayed and recorded in different assays along the wild-type (F397). Naturally occurring mutations F397Y, F397L, F397M, and F397I at site 397 of bacterial β-Glucosidase B expressed at higher levels (1.8 - 2.7 mg/mL) than any other amino substitution tested, and at similar level to the wild-type (F397, 3.7 mg/mL) ( **Fig. 1C, D** ), likely due to their similar physiochemical properties.&nbsp;

The expression pattern observed for substitutions at position 397 indicates that this site strongly favors hydrophobic and aromatic side chains. Aromatic residues (Y, W) showed the highest expression levels **( Fig. 1C, D)** , with hydrophobic aliphatic residues (A, V, I, L, M) showing moderately reduced but still substantial expression, suggesting that F397 contributes to a hydrophobic or aromatic core essential for proper BglB folding. In contrast, polar and charged substitutions markedly reduced expression **( Fig. 1D )** , consistent with the idea that introducing hydrophilicity or charge at this position destabilizes the protein and impairs folding (Laue, 2016). Charged mutations (D, E, K, R) were particularly detrimental, highlighting the sensitivity of this site to electrostatic disruption. Glycine and proline substitutions resulted in the lowest expression levels, likely due to their effects on backbone flexibility and secondary structure, with proline being especially disruptive to protein folding (Bajaj et. al. 2007).&nbsp;

Foldit standalone is a predictive software that allows the user to estimate protein energy scores before and after a mutational change, representative of changes in protein stability.&nbsp; A trend in expression across quartiles was revealed when F397 variants were grouped by their predicted stability rank based on Foldit energies ( **Fig. 1E** ): variants in higher stability quartiles tended to display greater expression than those in lower quartiles. Although substantial variability was observed within each quartile, several of the highest‑expressing variants clustered in the most stable group, while lower‑expressing variants were in the least stable quartiles. Tyrosine (Y) and isoleucine (I) substitutions exhibited expression levels substantially higher than those of other variants within the same Foldit energy quartile.

All twelve expressed mutants ( **Fig. 1C** ) were tested to assess the impact of the 19 amino acid substitutions on the enzyme–substrate binding affinity. The Michaelis–Menten constants (K _M_ ) obtained for the mutants ranged from 1.33 to 9.02 nM, values comparable to that of the wild-type enzyme (1.76 nM).&nbsp; These K _M_ values fall within the range of variability previously reported for BglB wild-type (Najam & Mayoral, 2024). Collectively, these results indicate that residue F397 contributes minimally to the binding affinity of the enzyme–substrate complex.

Of the twelve expressed mutants, four substitutions at position F397, methionine, isoleucine, tyrosine, and leucine, retained all (methionine) or most (isoleucine, tyrosine, and leucine) of the catalytic efficiency (k _cat_ /K _M_ ) observed for the wild-type enzyme ( **Fig. 1F** ). The k _cat_ /K _M_ value obtained for the wild-type fell well within the variability (53.69-197.96 nM ^-1^ /min ^-1^ ) reported for this enzyme by Najam & Mayoral (2024). None of the substitutions resulted in an improvement over the wild-type activity. In contrast, the valine substitution exhibited the most pronounced decrease in catalytic efficiency relative to the other variants, suggesting that this residue may disrupt optimal enzyme–substrate interactions. Although valine retains hydrophobic character, its smaller side-chain volume compared with leucine, isoleucine, and methionine may weaken local packing interactions or substrate positioning, thereby reducing catalytic efficiency.

Thermal stability assays showed that none of the expressed F397 variants exhibited a significant improvement over the wild-type enzyme, indicating that substitutions at this position do not reinforce the protein structure sufficiently to enhance resistance to thermal denaturation ( **Fig. 1G** ). Instead, mutations at F397 generally reduced thermal stability, suggesting that this residue mildly plays a stabilizing role in maintaining the native fold. The alanine substitution resulted in a T _50_ (39.1 ± 2.9 °C) most similar to that of the wild-type phenylalanine (40.4 ± 0.4 °C) ( **Fig. 1G** ), likely because alanine’s small, nonpolar side chain preserves local hydrophobic character without introducing charge, polarity changes, steric clashes, or new hydrogen-bonding interactions, despite the loss of the aromatic ring. In contrast, histidine resulted in the lowest thermal stability T _50_ = 35.1 ± 0.2 °C ( **Fig. 1G** ). Histidine can gain or lose a proton at physiological pH (Liao et al., 2013), its substitution at F397 likely introduces a partial positive charge that disrupts local hydrophobic packing or generates unfavorable electrostatic interactions. This interpretation is supported by the observation that other charged substitutions at this position similarly reduced thermal stability, highlighting the importance of maintaining a neutral, hydrophobic environment at F397.

This study systematically evaluated the functional and structural relevance of residue F397 in BglB by generating all 19 possible amino acid substitutions. The results demonstrate that F397 is moderately tolerant to substitutions, particularly to hydrophobic, neutral amino acids such as methionine, leucine, isoleucine, and tyrosine, which are also observed in naturally occurring β-glucosidases. These variants expressed at higher levels and retained catalytic efficiency and substrate binding comparable to the wild-type, reflecting a natural preference for hydrophobic, neutral side chains at this site. In contrast, charged or highly polar substitutions frequently failed to express or led to reduced stability, also highlighting the importance of maintaining a hydrophobic environment at F397 for proper protein folding and structural integrity. Kinetic analyses showed mutations at F397 minimally affected substrate binding (K _M_ ), while catalytic efficiency (k _cat_ /K _M_ ) was preserved only in substitutions with similar physiochemical properties. Thermal stability assays further indicated that substitutions at F397 generally reduce the enzyme’s resistance to denaturation. Alanine, with its small nonpolar side chain, most closely resembled the wild-type thermal profile, whereas histidine and other charged residues significantly decreased thermal stability, likely due to perturbation of hydrophobic interactions or introduction of electrostatic repulsion.

Our findings indicate that F397 contributes modestly to catalytic function but plays a more critical role in maintaining local structural stability. These insights into the structural and functional constraints of F397 provide a foundation for rational engineering of BglB and other β-glucosidases. Datasets like this one generated from systematic mutational studies can be valuable training material for emerging AI algorithms, improving the ability to predict structure-function relationships and guide protein design in silico.

## Methods

Nineteen BglB mutations were designed using Foldit Standalone (Kleffner et al., 2017), an interface to evaluate protein structure stability. Each mutant was assigned a unique total system energy (TSE) score and individual residue energy values after the application of both the minimization and repack/shake functions to the residue of interest and ~35 neighboring residues. A lower energy score meant a more stable protein. Jalview was used to generate and visualize a multiple sequence alignment of 50 bacterial BglB protein sequences retrieved from the NCBI database.&nbsp;

All 19 possible mutations were induced using Kunkel nucleotide-directed mutagenesis as previously described (Carlin et al., 2016). Kunkel method products were transformed into *E. coli* DH5α, and selected colonies were subject to a DNA plasmid mini prep (QIAprep Spin Miniprep Kit), and followed by Sanger sequencing at Eurofins Genomics in Louisville, KY. Sequences were verified for error free and the incorporating of the targeted mutation.

Verified plasmids were transformed into *E. coli* BL21(DE3) and selected colonies grew on LB/Kan liquid media at 37°C and 255 RPM. Isopropyl β-D-1-thiogalactopyranoside was used to induce expression. Cells were collected, lysed, and the BglB histidine tagged mutant proteins were purified using nickel-based immobilized metal affinity chromatography.

The expressed and purified wild-type and F397 mutants were then subjected to Kinetic and Thermostability assays using 4-nitrophenyl-β-D-glucopyranoside as substrate. Hydrolysis of the substrate was monitored at 420 nm.&nbsp;

Substrate concentrations 100, 33.33, 11.11, 3.70, 1.23, 0.41, 0.14, and 0 mM were tested against the wild-type (F397) and all F397 mutants that expressed. The kinetic data was fitted to the Michaelis-Menten model using a SciPy nonlinear curve fitting function. k _cat_ and K _M_ values were calculated for each variant and the catalytic efficiency (k _cat_ /K _M_ ) was reported.

The thermal stability for each mutant was determined using T _50_ , a temperature at which 50% of the protein denatures. Each mutant was incubated in a thermocycler for 30 minutes at temperatures ranging from 30°C to 50°C. A multiplate reader set to 420 nm was used to monitor the product released in the reaction.&nbsp;

All experiments were carried out in triplicates. The data obtained were uploaded to the D2D CURE database, that allowed to generate graphs for visual representation of K _M_ , k _cat_ /K _M_ , and T _50_ parameters.

## Reagents

**Table:** 

10 mM ATP	NEB
T4 Polynucleotide Kinase	NEB
T4 DNA Ligase Buffer	NEB
T7 Polymerase	NEB
dNTPS 25 mM	NEB
pET-29b + BglB variants	5,211 bp plasmid with F397 mutants inserted
β-glucosidase B (BglB) from *Paenibacillus polymyxa* .	1,377 bp gene
*E. coli* DH5α cells	Thermofisher
*E. coli* BL21 cells	Thermofisher
